# Rubicon is a critical regulator of immune barrier integrity and limits enteric pathogen infection

**DOI:** 10.3389/fimmu.2026.1889997

**Published:** 2026-10-19

**Authors:** Alexis D. Scaffidi, Salvatore J. Scaffidi, Jeremy Carling, Austin Fajfer, Mubashshir Ali, Ryosuke Ono, Addison Hilyard, Rachel E. Buksbaum, Vivek Kumar, Rohit Shukla, Sidharth Mishra, Shalini Jain, Hariom Yadav, Bradlee L. Heckmann

**Affiliations:** 1 USF Health Neuroscience Institute, Tampa, FL, United States; 2 Department of Molecular Medicine, Morsani College of Medicine, University of South Florida, Tampa, FL, United States; 3 Department of Neurosurgery, Brain and Spine, Morsani College of Medicine, University of South Florida, Tampa, FL, United States; 4 USF Center for Microbiome Research, USF Microbiome Institute, Morsani College of Medicine, University of South Florida, Tampa, FL, United States; 5 Center of Excellence for Aging and Brain Repair, Morsani College of Medicine, University of South Florida, Tampa, FL, United States; 6 Department of Medical Engineering – University of South Florida, Tampa, FL, United States

**Keywords:** bacterial infection, CASM, host defense, innate immunity, noncanonical autophagy, Rubicon, *Salmonella*

## Abstract

**Introduction:**

Intestinal immune barrier integrity is governed by coordinated epithelial renewal, junctional organization, and vesicular trafficking. Herein, we identify a previously undefined role for the noncanonical autophagy protein Rubicon in the maintence of the intestinal immune barrier. Rubicon-dependent vesicular pathways, including conjugation of ATG8 proteins to single membranes (CASM) function to support vesicle trafficking homeostasis.

**Methods:**

Mice with global deletion of Rubicon (Rubcn^-^/^-^) were used to evaluate impact on gut permeability using FITC-dextran, overall intestinal anatomy and histopathology, junction protein homeostasis, and pathogen response.

**Results and discussion:**

Loss of Rubicon led to impaired intestinal barrier function and increased intestinal permeability, supported by a 10-fold increase in circulating FITC-dextran. Rubicon-deficiency was also associated with multiple anatomical changes including elongation of the small and large intestines, shortened small-intestinal villi, epithelial hyperplasia, and lymphoid expansion, amongst others. Mechanistically, Rubicon-deficiency resulted in aberrant trafficking and mislocalization of key junctional proteins including E-cadherin and occludin. Furthermore, metagenomic analysis of the intestinal microbial community revealed broad restructuring of the intestinal microbiota compared to WT animals. Importantly, Rubicon-deficiency hypersensitized mice to enteric pathogen infection. Rubcn^-^/^-^ mice exhibited accelerated and worsened mortality in addition to systemic bacteremia following infection with *Salmonella Typhimurium*. Bacterial burden was significantly in multiple tissues including mesenteric lymph nodes, spleen, and liver, further supporting failure of the intestinal barrier following loss of Rubicon. Collectively, these findings identify Rubicon as a novel and foundational regulator of IEC vesicular processing, epithelial junctional organization, intestinal barrier function, and host defense beyond established roles in innate immune cells.

## 1 Introduction

Macroautophagy (henceforth autophagy) is a highly conserved process that delivers intracellular cargo to the lysosome for degradation and nutrient recycling, a key mechanism for maintaining cellular homeostasis. Autophagy is regulated by a diverse set of autophagy-related genes (ATGs) and proteins that function to orchestrate the biogenesis and maturation of double membrane structures, termed autophagosomes, which sequester and traffic intracellular cargo to the lysosome (1, 2).

The autophagy machinery and a select subset of the ATG proteins further function in related, yet distinct vesicle trafficking pathways whereby the microtubule-associated protein light chain 3 (LC3/ATG8) is conjugated to single membrane vesicles instead of double membrane autophagosomes, a process termed “conjugation of LC3/ATG8 to single membranes” (CASM) (3, 4). CASM internalizes and degrades extracellular cargo through phagocytic (5–8) and endocytic processes and facilitates intracellular vesicle trafficking (5, 9–11). The Run domain Beclin-1-interacting and cysteine-rich domain-containing protein, Rubicon (Rubcn), is an obligatory regulator of CASM, promoting its initiation through direct interaction with the class III phosphatidylinositol 3-kinase (PI3K) complex components VPS34 and BECN1 (5, 12–14). Additionally, the serine-rich domain of Rubcn binds to NOX2, propagating reactive oxygen species production, an essential byproduct of CASM and a required component for efficient phagosome and endosome acidification (15, 16).

In addition to Rubcn, ATG16L1, a member of the E3 ligase complex formed with ATG5 and ATG12 during canonical autophagy (17, 18), is likewise required for CASM. Once the E3 ligase complex is formed, LC3 is lipidated to a double or a single membrane vesicle during autophagy or CASM, respectively (5, 17). The C-terminal WD-40 domain of ATG16L1, containing 40 amino acids of β-transducin repeats (5), has a critical interaction with Vacuolar ATPase driving the acidification of the lysosome that degrades engulfed cargo (3, 19–21). Previous studies show Rubicon, and more broadly CASM-deficiency leads to hyperinflammation in the context of autoimmune disorders (22–24), neurodegeneration (9, 10, 25, 26), efferocytosis (27, 28), and a variety of infections (17, 29–31).

Mutations and polymorphisms, in ATG16L1 have been linked to human disease as both causative and risk factor genetic events. One such mutation, T300A in ATG16L1 increases susceptibility of individuals to the development of Crohn’s disease (32–35). Previous studies have shown that homozygous mutation of ATG16L1^T300A^ inhibits CASM (36, 37), increases pro-inflammatory cytokine production (17, 37), decreases cell antimicrobial peptide secretion (37, 38), and increases susceptibility to infections, like *Salmonella Typhimurium*, herein *S.t* (19, 37, 39, 40). While this mutation is detrimental for immune regulation within the intestine, our understanding of the interplay between CASM, intestinal barrier integrity, and immune homeostasis remains incomplete.

In addition to infection, Crohn’s disease, and more broadly inflammatory bowel disease (IBD) are chronic inflammatory disorders of the gastrointestinal tract, affecting millions of people worldwide (41–44). While IBD is a multifaceted disease, intestinal barrier integrity is a key marker for overall disease progression and severity of symptoms (42). Intestinal epithelial cells (IECs) maintain this integrity via paracellular junctions. Paracellular transport between IECs is a highly regulated process by which paracellular junction proteins (tight junctions, adherens junctions, and desmosomes) selectively allow solutes to cross from inside the intestine to the bloodstream (45). The intestinal barrier can be disrupted through a variety of different mechanisms, such as overall IEC integrity and regulation of tight junction proteins, whereby damage to the barrier can result in defective bodily homeostasis, increased risk of gastrointestinal disease, and importantly loss of barrier protection against pathogenic infections.

While the aforementioned ATL16L1 T300A mutation is clearly detrimental for immune regulation within the intestine, our understanding of the interplay between CASM, intestinal barrier integrity, and immune homeostasis remains incomplete. In addition to CASM regulating immune homeostasis, we further hypothesized that Rubicon and CASM could play a role in maintaining intestinal anatomical and microbial homeostasis via regulation of intestinal permeability as recently suggested (46). Herein, we investigated the role of CASM utilizing genetic manipulation of the key CASM regulator Rubicon, to evaluate the role of CASM in shaping intestinal barrier integrity and host-protection in response to *S.t* infection.

In brief, our results revealed that Rubicon is essential for maintaining intestinal barrier integrity by playing a critical role in regulating intestinal epithelial architecture, leading to maintenance of gut microbial homeostasis. Rubicon is required for efficient cellular turnover within the intestine, and when absent, leads to an accumulation of epithelial cells within intestinal villi. Rubicon limits gut dysbiosis, dissemination and increases survival during enteric *S.t* infection (47, 48). Data provided herein, support a mechanistic role of Rubicon and likely CASM in facilitating the trafficking and localization of junctional proteins including E-cadherin and occludin to the lateral IEC membrane – ensuring appropriate cell-to-cell interactions and barrier maintence. Cumulatively, this work identifies a novel role for Rubicon in maintaining intestinal villi architecture including junctional protein homeostasis, microbial homeostasis within the gut, and protection against enteric infections including *S.t* septicemia.

## 2 Materials & methods

### 2.1 Mice

The Rubcn knockout (Rubcn^-/-^) mice were previously established (49). SNP analysis shows that there is more than 97% similarity between the genotypes (9). Unless otherwise noted, all experiments were performed on mixed sex cohorts of mice 6–17 weeks old. The University of South Florida Institutional Animal Care and Use Committee approved all procedures in accordance with the Guide for the Care and Use of Animals. All mice were housed in specific-pathogen-free facilities, in a 12-hour light/dark cycle in ventilated cages, with chow and water supply ad libitum. Mice used for endpoint analyses were euthanized via CO2 asphyxiation in a 12in x 12in x 12in chamber with a CO2 flow rate of 11.5 L/min equating to a displacement of 40% in accordance with AVMA guidelines.

### 2.2 Gut permeability

Gut permeability was assessed by quantifying the mean fluorescence intensity of fluorescein isothiocyanate-dextran (FITC-Dextran) (Sigma). The mice were fasted for two hours prior to oral gavage of 100 µl FITC-dextran (200mg/ml stock). After 2 hours, blood was collected from the mice via submandibular bleed. The serum was collected by centrifuging the blood at 2,000 RPM for 10 minutes at 4°C. The serum was diluted 1:10 in 1xPBS. 100 µl of the diluted serum was placed into each well of a 96 well plate. The fluorescence intensity of FITC-Dextran was measured by a Cytation3 Imaging Reader with an excitation of 485 nm and an emission wavelength of 528 nm.

### 2.3 IEC Isolation & zymosan trafficking

IECs were isolated from mice of the indicated genotypes as reported previously (50). Isolation of IECs was achieved using CD326 EpCAM microbeads (Miltenyi Biotec) per the manufacturer’s instructions. Purified IECs were subsequently plated for downstream analyses.

For evaluating CASM/Rubicon-dependent trafficking, cells were exposure to red-fluorescently labeled zymosan particles (Abcam) and degradation was assayed by loss of fluorescence over time using a Biotek Cytation 5 fluorescent plate reader per the manufacturer’s instructions. Data a presented herein as a percent of zymosan fluorescence from t=0.

### 2.4 Fecal sample collection

Feces were collected from male mice between 7–10 AM. The mice were placed in a clean cage with no bedding at the bottom. 4–5 fecal pellets were collected per mouse and placed into a pre-cooled DNA/RNAase free 1.5mL tube using disposable forceps. Feces that came into contact with urine were discarded. The tubes were kept on dry ice while additional samples were collected and then immediately stored -80 °C until DNA extraction. DNA extraction was performed on the fecal samples following the QIAamp PowerFecal Pro DNA Kit (QIAGEN, 51804).

### 2.5 Metagenomic shotgun sequencing

Metagenomic shotgun sequencing was performed in a single batch as per the previously established protocol (51–53). Briefly, fecal DNA was extracted from 150 mg of mouse stool using the QIAamp PowerFecal Pro DNA Kit (Qiagen, USA) according to the manufacturer’s protocol. DNA concentration was measured with the Qubit dsDNA HS Assay Kit (Thermo Fisher Scientific, USA). For library preparation, 150 ng of DNA was processed with the Illumina DNA Prep (M) Tagmentation kit (Illumina, Inc., San Diego, CA, USA) following the manufacturer’s instructions. Each sample was assigned a unique IDT for Illumina-Nextera DNA UD Index. Sequencing was performed on the Illumina NextSeq 1000 platform using the NextSeq 1000/2000 P2 Reagent Cartridge (300 Cycles) v3 (Illumina, Inc., San Diego, CA, USA). Data were stored in BaseSpace and analyzed with bioinformatics pipelines, as detailed in the bioinformatics and statistical analysis section (53).

Shotgun sequencing data were analyzed using the Yet Another Metagenomic Pipeline (YAMP) workflow (54). YAMP uses bbmap tools for de-duplication, trimming, and decontamination of metagenomic sequences. FastQC was used to visualize both raw and quality-controlled reads. Metagenomic Phylogenetic Analysis (MetaPhlAn)4 was applied for taxonomic binning and profiling of microbial communities and their relative abundances (55). The MetaPhlAn database contains approximately 1.1 million unique clade-specific marker genes from about 100,000 reference genomes, including nearly 99,500 bacterial and archaeal genomes and approximately 500 eukaryotic genomes (56). This database enables unambiguous taxonomic assignments, accurate estimation of organismal relative abundance, and species-level resolution for bacteria, archaea, eukaryotes, and viruses.

### 2.6 Tissue lysate and immunoblot

Intestines were homogenized in 10x weight of sample using RIPA buffer [50 mM Tris (pH 7.5), 150 mM NaCl, 1% Triton X-100, 0.5% deoxycholate (DOC), 0.1% SDS, protease inhibitor tablet (Roche), 1 mM NaF, 1 mM Na3VO4, and 1 mM PMSF] using a bead homogenizer. The samples were normalized to using a BCA (Thermo Fisher 23227) following manufacturer’s protocol. 4x sample buffer with 10% beta-Mercaptoethanol was added to samples. The lysates were boiled at 65 degrees Celsius and centrifuged for 10 minutes. 15ug protein was loaded into each 4-20% SDS-PAGE gel unless otherwise stated. The gel was transferred to PVDF membrane, blocked for 1 hour with 5% milk in TBS-T. Primary antibodies were added in 1% milk overnight at 4 degrees Celsius, secondary were added for 1 hour in 1% milk. The proteins were visualized using Clarity Western ECL substrate (Biorad #170-5061) or SuperSignal West Atto Ultimate Chemiluminescent substrate (Thermo scientific A38555). Densitometry was performed using ImageJ. The primary antibodies used for microscopy were as follows: caspase 3 (Cell Signaling, 9662S), cleaved caspase-3 (D175) (Cell Signaling 9661S). The secondary antibodies used were as follows: anti-rabbit IgG HRP-linked antibody (Cell signaling 7074S), anti-rat IgG HRP-linked antibody (Cell Signaling 7077S).

### 2.7 Microscopy and image analysis

Intestines were isolated from mice post CO_2_ asphyxiation as described above and cervical dislocation per our IACUC protocol. The intestines were flushed with 3 mL cold 1xPBS and placed in 4% paraformaldehyde for 24 hours. Following fixation, they were dehydrated using 30% sucrose and then embedded in OCT. All sections were performed using a cryotome (Thermo) at 10 µm, unless otherwise stated. All samples underwent antigen retrieval in 1x citrate buffer and blocked in 5% BSA, 0.1% triton-x 100 for 1 hour. For cell-based imaging cells were fixed in 4% paraformaldehyde for 10 minutes at room temperature and subsequently washed 3x in 1xPBS. Cells were then permeabilized in 0.1% Triton-X 100 in 1xPBS for 5 minutes followed by washing in 1xPBS. Cells were blocked in 1xPBS containing 0.5% BSA for 30 minutes prior to incubation with primary antibodies as described below.

Primary antibodies were added at a 1:200 dilution in blocking buffer overnight at 4 degrees C and secondary antibodies were added at a 1:300 dilution in blocking buffer for 2 hours at room temperature. Images were taken using Nikon TI eclipse confocal microscope. Images were analyzed using ImageJ unless otherwise noted. The primary antibodies used for microscopy were as follows: Na, K-atpase (Cell Signaling, 3010s), caspase 3 (Cell Signaling, 9662S), cleaved caspase-3 (D175) (Cell Signaling 9661S), Lysozyme (Abcam, EPR2994(2)), LC3/ATG8 (Cell Signaling, 4108s), occludin (Cell Signaling, 68534). The following secondary antibodies were used as follows: Alexa Fluor 488 AffiniPure Donkey Anti-Rat IgG (H+L) (Jackson ImmunoResearch 712-545-150) and Alexa Fluor 594 AffiniPure Donkey Anti-Rabbit IgG (H+L) (Jackson ImmunoResearch 711-585-152). DAPI was stained using either ProLong Gold Mountant with DAPI (Invitrogen, P36931) for tissue sections or ProLong Gold Reagent with DAPI (Invitrogen, P36975).

### 2.8 *Salmonella typhimurium* infection and CFU/mL quantification

*Salmonella enterica* serovar Typhimurium (*S. typhimurium, S.t*) (ATCC 14028) was used to infect mice for survival curve and CFU/mL experiments. For the survival curve, mice were orally infected with 1x10^9^ CFU/mL and monitored for up to 7 days. The mice were euthanized when they reached endpoint (predefined as hunching, a rough coat, greater than 20% weight loss, and any significant fatigue) or at the end of 7 days.

To quantify the CFU/mL of the infected organs, we infected mice with 1x10^9^ CFU/mL *S.t* and euthanized and collected the organs 3 days post infection (DPI) on ice. The organs were homogenized in 2x volume of sterile 1x PBS for 3 minutes using a bead beater (Benchmark D1030 BeadBug 3). The lysates were diluted with sterile 1x PBS serial diluted from 10–^1^ to 10^-8^. 30 µl of undiluted lysate and the diluted lysates were plated and incubated at 37°C overnight. The following morning colonies were counted, and the CFU/mL was calculated using the following formula: (number of colonies x dilution)/volume plated.

### 2.9 BrdU incorporation and migration assay

Mice were oral gavaged with 10mg/kg 5-Bromo-2′-deoxyuridine (BrdU) (Sigma-Aldrich, 59-14-3). Mice were humanly euthanized 24–48 hours post-exposure using CO2 asphyxiation as described above and cervical dislocation as a secondary measure. The ileum was collected, flushed with cold PBS, cut in half, and washed again with cold PBS in a petri dish. The cleaned ileum was then opened with the villi side facing up and rolled into a swiss roll proximally to distally, as previously described (57). The swiss roll was then transferred to a cassette and placed in 4% PFA. 24 hours later, the cassette with the fixed ileum was transferred to 30% sucrose for 24 hours. The sample was then embedded and froze OCT after drying the sample with a kimwipe. Each sample was sectioned into 10 µm sections using a cryotome (Thermo).

Once the samples were sectioned, the tissue underwent immunofluorescence staining as described in “microscopy and image analysis” using BrdU primary antibody (Anti-BrdU, Abcam, ab6326) and a 488-secondary antibody. Following imaging, the samples were analyzed using imageJ and QuPath. We based our analysis on the following paper (58). In short, we counted the number of BrdU-positive (BrdU+) cells per villi using ImageJ to measure cell proliferation rates. We subsequently measured the distance of the furthest BrdU+ cell and divided that by the villi length to quantify the distance traveled by the proliferating cells, measuring cell migration.

### 2.10 Measuring the length of intestines

Mice were humanely euthanized as described above by CO2 asphyxiation with a secondary cervical dislocation. The intestines were collected and unwound prior to being flushed clean and the length was measured using a ruler with the centimeter markings.

### 2.11 Intestine tissue fractionation

To determine the abundance of tight junction proteins within cellular compartments, we utilized tissue fractionation of intestine samples into cytosol (C) and plasma membrane (PM) fractions, using the Minute™ Plasma Membrane/Protein Isolation and Cell Fractionation Kit (Invent Biotechnologies SM-005). Briefly, 20–30 mg pieces were cut from the ileum of intestine samples and homogenized for 1 minute in the Invent filter cartridge with 200 μL Buffer A. Once homogenized, 300 μL of Buffer A was added and the tube was incubated on ice for 5 minutes. To separate the supernatant from any unhomogenized sample, the tube was centrifuged at 16,000 X g for 30 seconds. The pellet was then quickly vortexed, and the sample was centrifuged at 700 X g for 1 minute, to pellet out the nuclei fraction. The supernatant was then transferred into a new tube and centrifuged at 16,000 X g for 30 minutes at 4 °C, to pellet a total membrane fraction. Again, the supernatant, which contains the cytosolic fraction, was transferred to new tube, and the remaining pellet was resuspended in 200 μL of Buffer B. Once resuspended, tubes were centrifuged at 8,000 X g for 5 minutes at 4 °C. The supernatant, which contain the plasma membrane fraction, was transferred to a tube containing 1.2 mL of cold PBS and vortexed to mix. These tubes were then centrifuged at 16,000 X g for 30 minutes at 4 °C. The pelleted plasma membrane fraction was then resuspended in 200 μL of Minute™ Non-Denatured Protein Solubilization Reagent (Invent WA-010) with 1% SDS added. Total protein concentrations of the cytosolic and plasma membrane fractions were quantified using a BCA. Normalized samples were then run on SDS-PAGE for subsequent immunoblotting for E-cadherin (Cell Signaling 3195T), Na, K-ATPase (Cell Signaling 3010S) and β-Actin (ProteinTech HRP-60008).

### 2.12 Histology

Small and large intestines were processed into swiss rolls as previously described (57) and fixed with 10% formalin for 2 days. The formalin fixed swiss rolls were sent to Moffitt cancer center histology core for full processing and analysis. This included paraffin embedding, sectioning, H&E staining, and analysis. The sections were 5 µm thick and the H&E staining was performed using a pre-optimized automated protocol. Analysis was performed as previously described (59). Length of villi was assessed using the measure tool on QuPath (version 0.5.1-64x). A minimum of 50 villi were counted per sample.

### 2.13 Quantification protocol for membrane-associated junctional proteins

To assess the localization of junctional proteins within intestinal villi, we quantified the total fluorescence across the width of individual villi and compared WT to Rubcn-/-. Using ImageJ, a line was drawn across the width of a cell, and a multi-plot analysis of the fluorescence curve was calculated. Relative fluorescence values were collected, across 50 cells per section with 3 sections per animal/genotype, and plotted on the y-axis against the cell diameter on the x-axis. Graphed lines show the average fluorescence at a given location within a cell.

### 2.14 Quantification and statistical analysis

Data are presented as the mean ± standard error of the mean (SEM), unless otherwise indicated. Individual data points represent independent biological replicates, and sample sizes are reported in the corresponding figure legends. Technical replicates, when applicable, were averaged to generate a single value for each biological replicate. Comparisons between two independent groups were performed using two-tailed unpaired Student’s t-tests. P-values less than 0.05 were considered significant. All statistical analyses were performed using GraphPad Prism 10.2.0 software. To analyze images, we utilized either ImageJ (version 1.53t) or QuPath (version 0.5.1-64x) software.

## 3 Results

### 3.1 Rubicon is a critical regulator of intestinal anatomy and permeability

The maintenance of intestinal barrier function is paramount to limit chronic inflammation and prevent a variety of diseases, such as diabetes, cardiovascular disease, and neurodegenerative disease (60). When intestinal barrier function is disrupted, harmful agents such as bacteria, viruses, and microbiota metabolites, among others leak into the blood stream unregulated, canonically termed “leaky gut syndrome” (60).

To assess the impact of CASM on intestinal permeability, we analyzed gut permeability using FITC labeled-dextran in CASM-deficient mice (Rubcn^-/-^). We evaluated dextran leakage from the intestine to the blood over a 2-hour period following oral administration of the labeled dextran. Wild-type littermate control (WT) mice showed a relatively low, basal level of gut permeability with detected FITC-fluorescence around 1 AU. In contrast, Rubcn^-/-^ mice had an approximate 10-fold increase in levels of FITC-dextran within the blood 2 hours post-administration with levels reaching 10 AU and higher (**Figure 1A**). These data suggest that CASM-deficiency increases gut permeability and support the hypothesis that CASM is critical for maintaining gut integrity.

**Figure 1:**
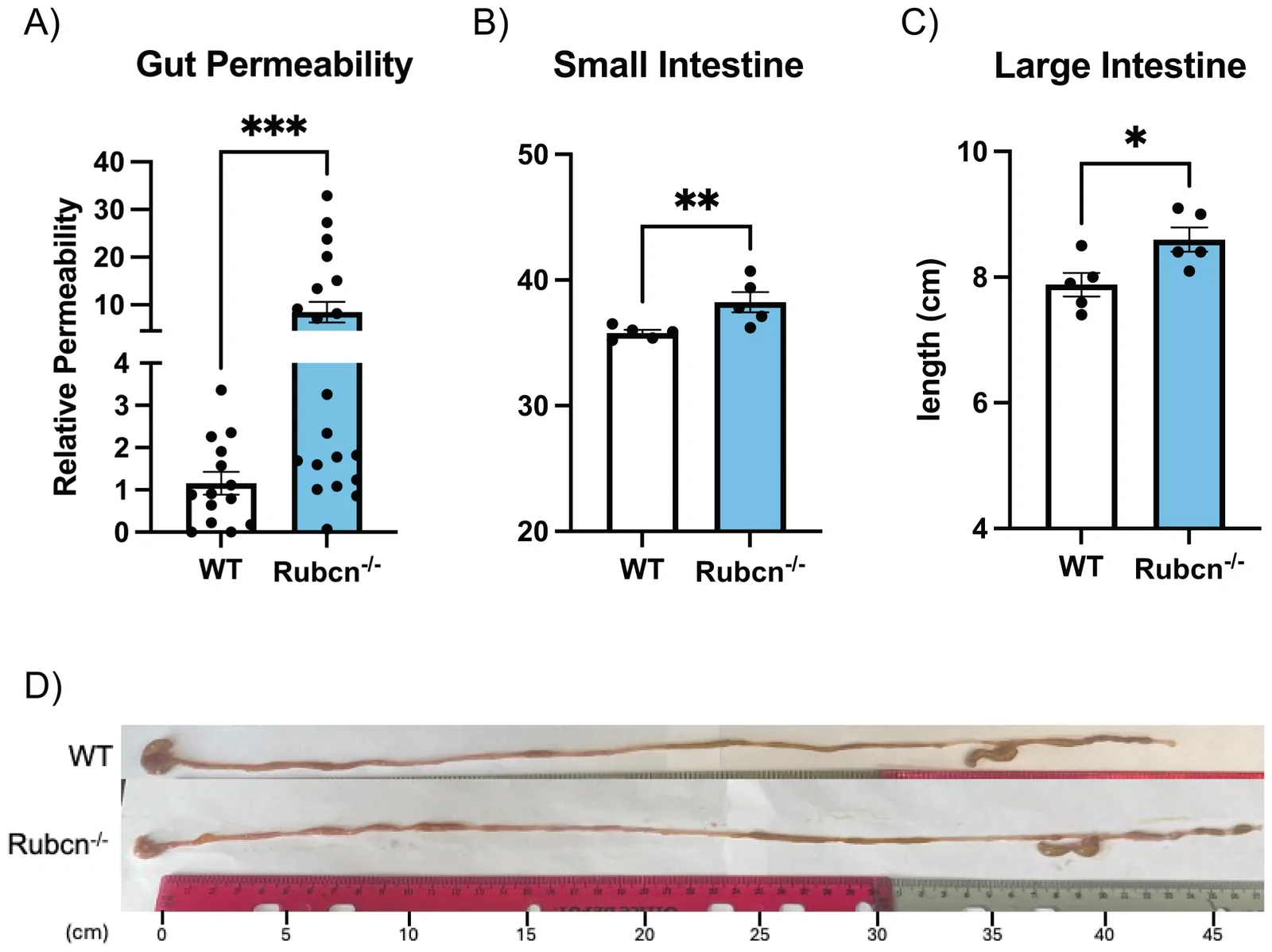
Rubicon is essential for maintaining paracellular integrity. **(A)** Levels of 4kda FITC-dextran in serum collected 2 hours post-administration in 6-14-week-old C57Bl/6 (WT) or Rubcn^-/-^ mice. For WT, *n=*14, for Rubcn^-/-^ *n* = 26 mice, each dot represents an individual animal. **p=0.008. **(B)** Quantification of small intestine length of WT and Rubcn^-/-^ mice. Each dot represents an individual mouse. *n=*5 per genotype. **p=0.0099. **(C)** Quantification of large intestine length of WT and Rubcn^-/-^ mice. Each dot represents an individual mouse. *n=*5 per genotype. *p=0.0141. **(D)** Representative image of WT and Rubcn^-/-^ intestines. Data are represented as mean ± SEM. Significance was calculated using a two-tailed unpaired Student’s t-test.

ATG16^T300A^, a mutation which limits CASM (29, 36), enhances susceptibility to Crohn’s disease (36, 37). Previous studies have shown that mice deficient in ATG16L1 have decreased colon and villi lengths (61) due to an increase in immune cell activation, inflammation, and Paneth cell dysfunction (62). We first assessed Paneth cells in Rubcn^-/-^ ileum by quantifying the number of lysozyme-positive cells within small intestinal crypts and the abundance of lysozyme being expressed. We hypothesized that the Rubcn^-/-^ ileum would have a reduction in Paneth cell number and lysozyme expression consistent with ATG16L1-deficient mice when compared to WT ileums. Surprisingly, we saw no difference between Rubcn^-/-^ and WT Paneth cells number or expression of lysozyme (**Supplementary Figures S1A, B**). We then broadened our scope to determine whether there was a change in the overall length of the Rubcn^-/-^ intestines (**Figure 1D**). Our results revealed a significant increase intestinal length in both the Rubcn^-/-^ small (**Figure 1B**) and large (**Figure 1C**) intestines. Taken together these data suggest that in addition to restricting gut permeabilization, CASM is influencing the anatomical organization of both small and large intestine.

### 3.2 Rubicon-deficiency leads to altered epithelial junctional organization

Given the increased intestinal permeability observed in the Rubcn^-/-^ mice, we next investigated whether Rubicon-deficiency affected the organization of key epithelial junctional proteins. E-cadherin and occludin are key components of adherens and tight junctions respectively, and function to ensure appropriate cell-cell contacts to regulate barrier permeability (63, 64).

Although there were no overt alterations in the level of E-cadherin in the ileum of WT compared to Rubcn^-/-^ mice, there was a robust change in the membrane association of E-cadherin between the genotypes. Consistent with its function, E-cadherin exhibited prominent localization along the lateral epithelial membrane in WT animals, whereas in Rubcn^-/-^ mice, E-cadherin displayed a less organized membrane distribution with increased intracellular signal (**Figures 2A, B**). These findings were further supported by biochemical fractionation demonstrating clear increases in cytosolic E-cadherin in the Rubcn^-/-^ mice compared to their WT counterparts which had an approximate 4x increase in E-cadherin found in the membrane fraction compared to the cytosol (**Figures 2C, D**).

**Figure 2:**
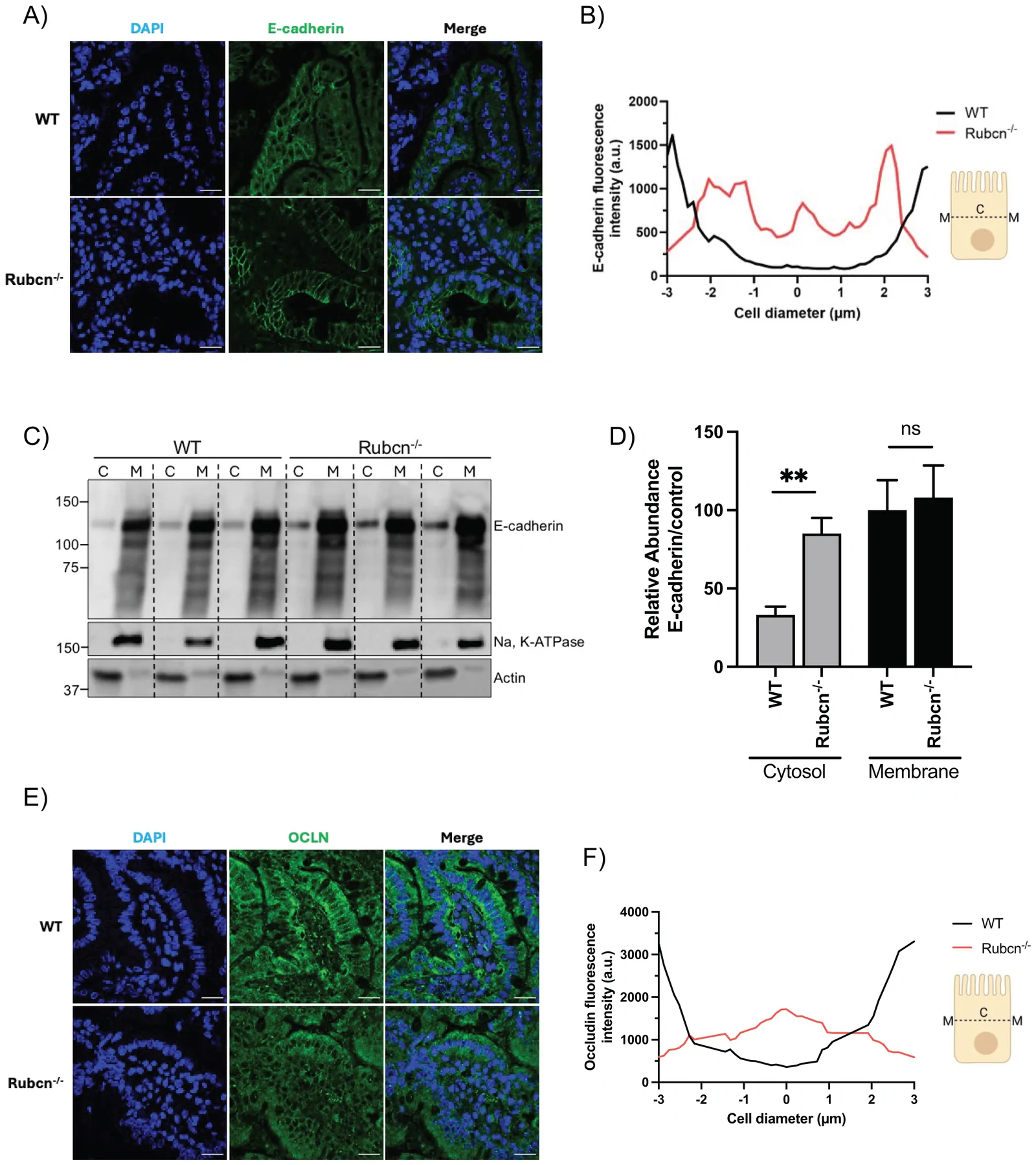
Rubicon-deficiency leads to altered epithelial junctional organization. **(A)** Representative images showing E-cadherin localization in WT and Rubcn^-/-^ ileum in 10-week-old mice. Scale bar, 20 μm. **(B)** Membrane vs. cytosolic distribution analysis of E-cadherin in WT and Rubcn^-/-^ ileum in 10-week-old mice. 50 cells/section were quantified for 3 sections per genotype and averaged for *n=*5 mice per genotype. **(C)** Immunoblot analysis for E-cadherin distribution in cytosol and membrane fractions in ileum from 10-week-old WT and Rubcn^-/-^ mice. *n=*3 mice/genotype, Actin was used as a cytosolic marker and Na,K-ATPase used as a membrane marker. **(D)** Quantification of immunoblot analysis presented in panel **(C)**. Presented as relative abundance of E-cadherin normalized to the respective fraction control. **p=0.0091. **(E)** Representative images showing Occludin (OCLN) localization in WT and Rubcn -/- ileum in 10-week-old mice. Scale bar, 20um. **(F)** Membrane vs. cytosolic distribution analysis of Occludin in WT and Rubcn -/- ileum in 10-week-old mice. 50 cells/section were quantified for 3 sections per genotype and averaged for n=5 mice per genotype. Data are represented as mean ± SEM. Significance was calculated using a two-tailed unpaired Student’s t-test.

Consistent with our findings on E-cadherin, occluding was likewise disproportionately distributed in the Rubcn^-/-^ mice. WT animals exhibited clear membrane localization of occludin with little intracellular signal being detected, whereas Rubcn^-/-^ mice had a diffuse occludin organization with prominent intracellular signal (**Figures 2E, F**).

These findings indicate that Rubicon supports the appropriate subcellular organization of both adherens- and tight-junction proteins and is suggestive of dysfunctional junctional protein localization that may contribute to the increased intestinal permeability observed in the Rubcn^-/-^ mice.

### 3.3 Rubicon promotes LC3 localization to occludin-containing vesicles in intestinal epithelial cells

Rubicon has been shown to be a critical regulator of intestinal epithelial cell (IEC) function in the context of CASM-mediated internalization and trafficking of pathogens including luminal fungi (65). Furthermore, CASM is critical for the appropriate trafficking of multiple immune sensors and signaling proteins including toll-like receptor 4 (TLR4) and CD36 (9, 14). As such, we hypothesized that Rubicon-dependent vesicle trafficking may be critical for the distribution of junctional proteins, in particular occludin to the lateral membrane given the clear mislocalization observed in the Rubcn^-/-^ mice.

To investigate this further we evaluated Rubcn^-/-^ IECs and first confirmed alterations in Rubicon-dependent CASM-mediated trafficking. Cells were exposed to fluorescently labeled zymosan particles and degradation was monitored longitudinally as reported previously (9, 14). Consistent with a defect in CASM, Rubcn^-/-^ IECs exhibited delayed degradation of zymosan compared to WT IECs (**Supplementary Figure S2**).

Immunofluorescence analysis demonstrated prominent peripheral occludin staining in WT IECs and highly central localization in Rubcn^-/-^ IECs (**Figure 3A**). Interestingly, co-staining with LC3 revealed a clear and consistent organized LC3+ vesicle structure in the WT IECs that in contrast was highly fragmented in the Rubcn^-/-^ IECs (**Figures 3A, B**). Furthermore, WT IECs had appreciable numbers of LC3+ vesicles containing occludin, whereas these vesicles were robustly decreased and, in many cases, absent in the Rubcn^-/-^ IECs (**Figures 3B, C**).

**Figure 3:**
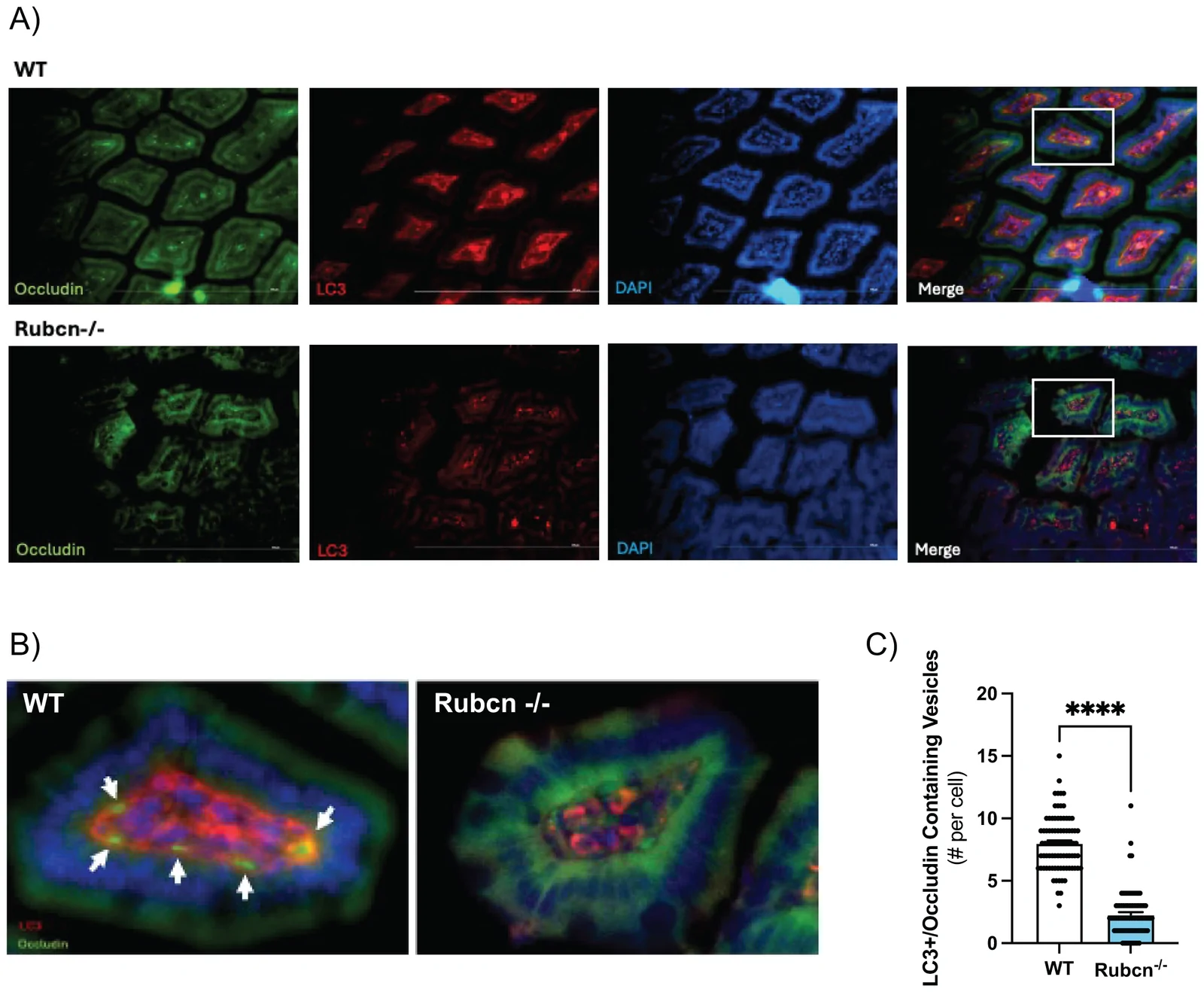
Rubicon promotes LC3 localization to occludin-containing vesicles in intestinal epithelial cells. **(A)** Representative imaging of WT and Rubcn^-/-^ IECs stained for occludin and LC3. **(B)** Higher magnification imaging of the selected IECs boxed in panel **(A)**. White arrows indicate LC3+ vesicles containing occluding. (Occludin – Green, LC3 – Red, DAPI – Blue). **(C)** Quantification of the number of LC3+/occludin-containing vesicles per IEC. 85 cells were analyzed per genotype. Data are presented as mean ± SEM. Each dot represents a single cell measured. Significance was calculated using a two-tailed unpaired Student’s t-test. ****p=0.0001.

These results strongly support a critical role for Rubicon-dependent CASM-mediated vesicle trafficking in facilitating the proper localization of junctional proteins such as occludin, as Rubicon-deficiency limited the association of LC3+ vesicles with occludin and resulted in more central occludin localization compared to WT IECs.

### 3.4 Rubicon functions to maintain healthy IEC architecture

The intestinal epithelium is highly organized and tightly regulated by various mechanisms, including Wnt signaling, extracellular matrixes, and mechanical forces (66). To gain a more detailed understanding of the broader intestinal anatomy in the context of Rubicon-deficiency, we performed histological analysis of Hematoxylin and Eosin (H&E) stained small and large intestines (**Figure 4A**). For histological analyses, we utilized a standardized histological pathology scoring system of 0-5 (59), where 0 represents “none present” and 5 representing the highest “marked” severity (**Figure 4B**).

**Figure 4:**
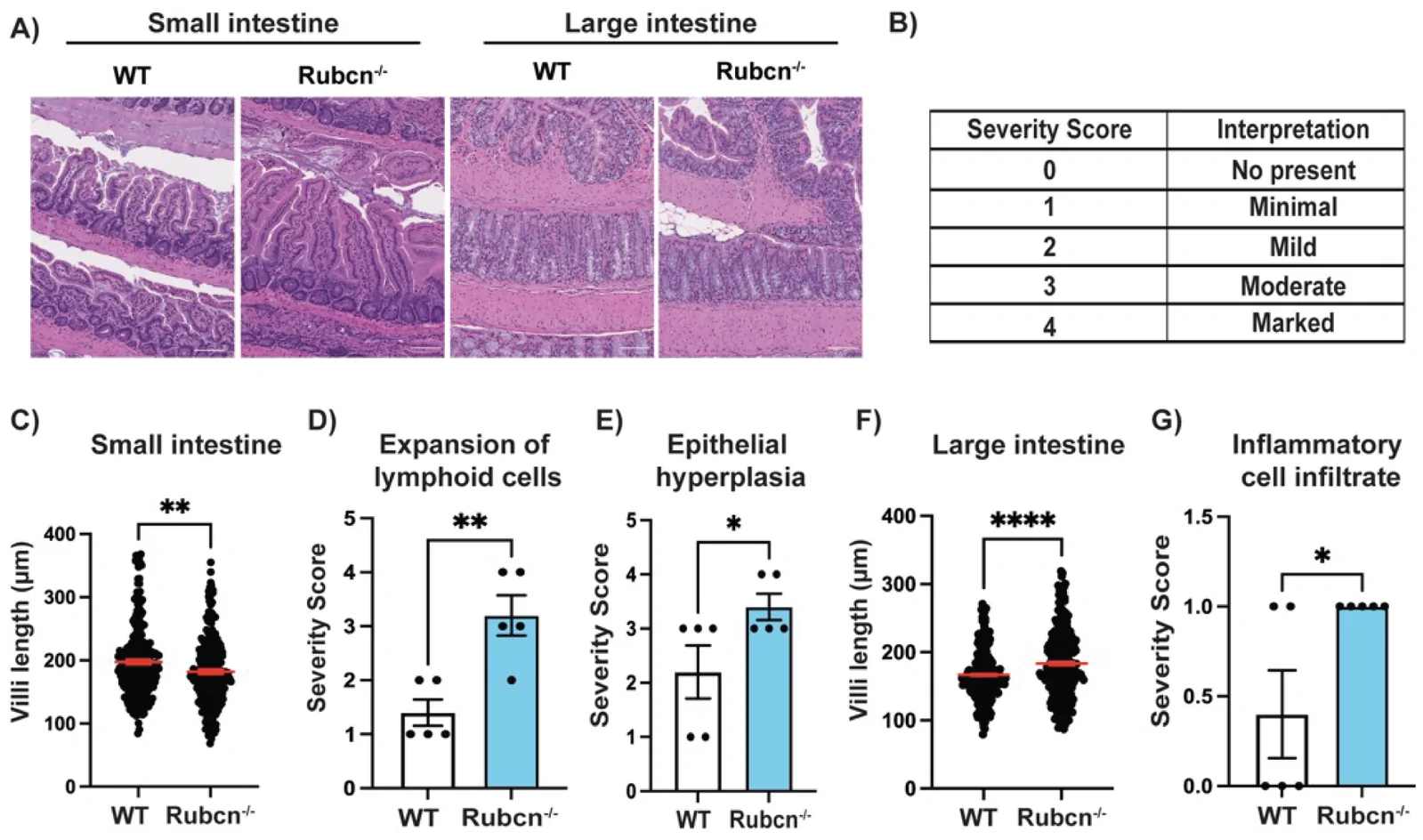
Rubicon-deficiency disrupts intestinal architecture. **(A)** Representative images of small and large intestine stained for H&E from 10-week-old WT and Rubcn^-/-^ mice. **(B)** Severity score system used for analysis of H&E images. **(C)** Quantification of small intestinal villi length. Each point represents a villus. *n=*258 WT villi and *n=*249 Rubcn^-/-^ villi across 3 independent sections/mouse from 5 mice per genotype. **p=0.0026. **(D, E)** Histological quantification of **(D)** expansion of lymphoid cells and **(E)** epithelial hyperplasia within the small intestine. *n=*5 mice per genotype and each dot represents an individual animal. **p=0.0019 for **(D)** and *p=0.0299 for **(E)**. **(F)** Quantification of large intestinal crypt length. Each point represents a crypt. *n=*257 WT crypts and *n=*253 Rubcn^-/-^ crypts across 3 independent sections/mouse from 5 mice per genotype. ****p=0.0001. **(G)** Histological quantification of infiltration of lymphoid cells within the large intestine. *n=*5 mice per genotype and each dot represents an individual animal. *p=0.02. Data are represented as mean ± SEM. Significance was calculated using a two-tailed unpaired Student’s t-test.

Measuring the length of intestinal villi can be used as a tool to measure the overall health of intestines. WT small intestinal villi typically measure around 200–250 microns in length (67–69), with shorter villi indicating a more inflamed, unhealthy intestine (68). We observed significantly shortened villi in the Rubcn^-/-^ small intestine compared to WT (**Figure 4C**). Interestingly, the Rubcn^-/-^ small intestine also showed moderate to severe expansion of lymphoid cells compared to WT small intestine, which as expected showed minimal expansion of these cells (**Figure 4D**). The Rubcn^-/-^ small intestines also had a moderate to severe epithelial hyperplasia, whereas WT small intestines only had mild epithelial hyperplasia (**Figure 4E**). Conversely, there was no significant difference among any of the other categories we analyzed, including goblet cell loss and crypt loss (**Supplementary Figures S3A–J**). Shortened villi (68), increased lymphoid cell expansion, and epithelial cell hyperplasia (69) are all consistent with Crohn’s Disease models and increased inflammation.

In addition to the small intestine, we likewise evaluated architectural changes in the large intestine, specifically the colon. While large intestines do not have classical villus structures, we measured the colonic crypt length. The expected healthy length of colonic crypts in mouse large intestine is approximately 150 microns (70). In contrast with the small intestine, the Rubcn^-/-^ large intestinal crypts were significantly longer than the WT (**Figure 4F**). The histological analysis of Rubcn^-/-^ large intestine revealed a significant increase in inflammatory cell infiltration (**Figure 4G**). However, proliferation of lymphoid cells was not as marked as observed in the small intestine (**Supplementary Figure S3K**). Overall, we observed key differences in the architecture and cellular landscape of both the small and large intestine in Rubcn^-/-^ mice.

Together, these results indicate a role for Rubicon in regulating inflammatory cell infiltration and proliferation within the intestine, along with maintenance of IEC proliferation specifically within the small intestine.

### 3.5 Rubicon is an essential regulator of IEC villi turnover

The intestine is a very harsh environment with mechanical, chemical, and biological stressors, which require the intestine to constantly regenerate the villi with new cells to maintain homeostasis. Due to the altered length of the intestine in our Rubicon-deficient model, we evaluated small intestinal villi architecture by staining with a plasma membrane marker, sodium potassium ATPase (Na, K-ATPase). In WT mice, the small intestinal villi are characterized by an organized linear anatomy with cells appearing in a ladder-like structure. Interestingly, we observed stacked cells that were intrinsically disordered within the Rubcn^-/-^ villi (**Figure 5A**). IECs have a turnover rate of approximately 3–5 days (71). If this mechanism becomes disrupted, it is possible to increase the accumulation of cells within the villi, as shown in **Figure 5A**. We hypothesized that there is a decrease in cellular turnover within the Rubcn^-/-^ villi, therefore we performed BrdU incorporation and cell migration analyses. At 24-hours post-BrdU exposure, we observed a significant increase in the number of BrdU-positive cells per villi in the Rubcn^-/-^ ileum (**Figure 5C**). We also measured the absolute distance travelled for BrdU+ cells and observed a significant increase in the Rubcn^-/-^ villi within the ileum (**Figure 5D**). When we normalized to villi length, we still observed this same increase despite varying villi lengths (**Figure 5E**). At 48-hours post-BrdU exposure, there was no significant difference in the number of BrdU-positive cells (**Supplementary Figure S4B**), but similarly we detected an increase in both absolute distance travelled of BrdU-positive cells and distance travelled when normalized to villi length, (**Supplementary Figures S3C, D**).

**Figure 5:**
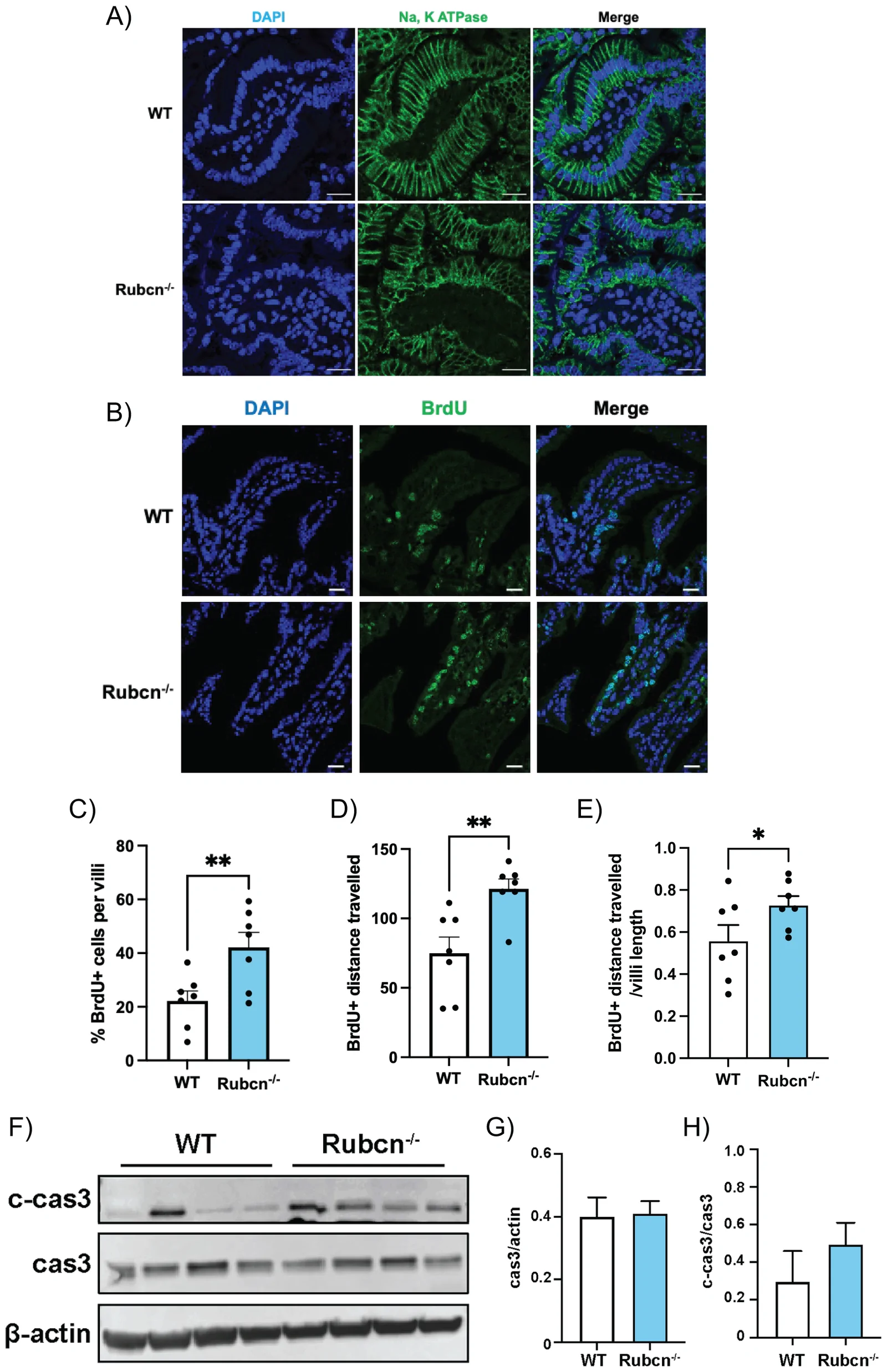
Rubicon is required for organizing intestinal epithelial cells in villi. **(A)** Representative imaging of the ileum plasma membrane from WT and Rubcn^-/-^ 10-week-old mice. Scale bar, 20 μm. **(B)** Representative imaging of the BrdU incorporation within the ileum of 15–17-week-old WT and Rubcn^-/-^ mice. Scale bar, 20 μm. **(C)** Quantification of the number of BrdU-positive cells per villi in each image. *n=*3 slides/animal from 7 mice per genotype. **p=0.0065. **(D)** Quantification of the absolute distance of the furthest BrdU-positive cell in each image. *n=*3 slides/animal from 7 mice per genotype. **p=0.005. **(E)** Quantification of the distance of the furthest BrdU-positive cell in each image normalized to the length of the villi. *n=*3 slides/animal from 7 mice per genotype. *p=0.039. **(F)** Immunoblot of total caspase 3 (cas3), cleaved-caspase 3 (c-cas3), and actin within the ileum of 11-14-week-old WT and Rubcn^-/-^ mice. **(G, H)** Densitometry of immunoblot measuring cleaved-caspase 3 over total caspase 3. Data are represented as mean ± SEM. Significance was calculated using a two-tailed unpaired Student’s t-test.

Cellular turnover in the gut typically proceeds through canonical apoptosis. To measure apoptosis levels within the Rubcn^-/-^ ileum, we performed a western blot to determine the overall abundance of pro-caspase 3 (casp3), and its active form, cleaved caspase 3 (c-casp3) (**Figure 5F**). As expected, we detected no difference in casp3 abundance (**Figure 5G**), and while not significant, we observed an increase in c-casp3 in the Rubcn^-/-^ ileum compared to the WT control when normalized to total casp3 levels (**Figure 5H**). These results are consistent with a potential increase in apoptotic activity within the Rubcn^-/-^ ileum, leading us to hypothesize that the Rubcn^-/-^ IECs within the ileum attempt to overcome increased cell death by enhancing proliferation and migration to normalize gut homeostasis.

### 3.6 Rubicon supports the maintenance of intestinal microbiome homeostasis

Since increases in gut permeability are highly correlated to intestinal dysbiosis (72, 73), we performed metagenomic analysis of the microbiome and found Rubcn-deficiency robustly altered the gut microbial profile when compared with WT controls. At the genus level, Rubcn^-/-^ mice showed significantly reduced relative abundance of *Prevotella* and *Bacteroides*, together with increased abundance of *Acutalibacter, Oscillibacter, Acetatifactor*, and unclassified *Firmicutes* (**Figures 6A–G**). Viral genera were also significantly altered in Rubcn^-/-^ mice, including *Betaretrovirus* and *Gammaretrovirus*, which were decreased (**Figures 6H–J**). Family-level analysis supported these findings, with increased *Muribaculaceae*, *Ruminococcaceae*, and *Oscillospiraceae* and reduced *Prevotellaceae* in the knockout mice (**Supplementary Figure S5**). Species-level analysis further identified increased *Acetatifactor muris*, *Oscillibacter* sp. *1_3*, *Acutalibacter muris*, and *Firmicutes bacterium ASF500*, alongside decreased *Prevotella* sp. *MGM2* and *Bacteroides caecimuris* (**Supplementary Figure S5**). Together, these data indicate that Rubcn-deficiency, and likely the alterations in intestinal permeability as a result, are associated with broad restructuring of the intestinal microbiome.

**Figure 6:**
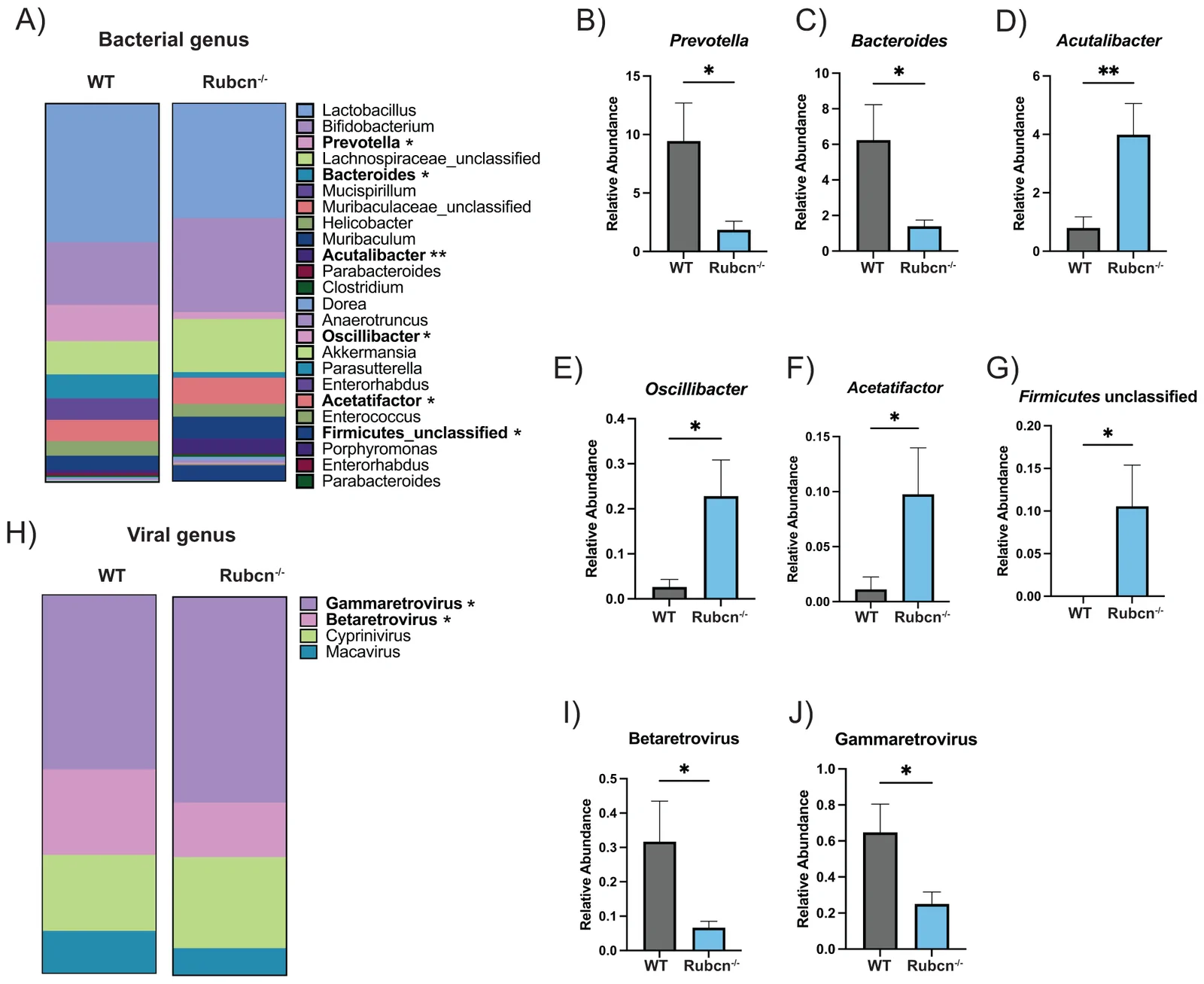
Rubicon-deficiency drives intestinal dysbiosis. **(A)** Quantification of bacterial genus in WT and Rubcn-/- mice. Whole genome sequencing was performed on fecal samples of 5 mice per genotype. Data are represented as parts of a whole before any outliers are removed. Significance was calculated using unpaired t-test for each genus. ∗p < 0.05, ∗∗p < 0.01, ∗∗∗p < 0.001. **(B–G)** Quantification of bacterial genus populations with significant differences between WT and Rubcn-/- samples. *n=*5 mice per genotype. Outliers were calculated using ROUTs method. Significance was calculated using unpaired t-test for each genus. For **(B)** *p=0.0336, **(C)** *p=0.0286, **(D)** **p=0.007, **(E)** *p=0.0198, **(F)** *p=0.0419, and **(G)** *p=0.0489. **(H)** Quantification of viral genus in WT and Rubcn-/- mice. Whole genome sequencing was performed on fecal samples of 5 mice per genotype. Data are represented as parts of a whole before any outliers are removed. Significance was calculated using unpaired t-test for each genus. ∗p < 0.05. **(I, J)** Quantification of viral genus populations with significant differences between WT and Rubcn-/- samples. *n=*5 mice per genotype. Outliers were calculated using ROUTs method. Significance was calculated using unpaired t-test for each genus. For **(I)** *p=0.045 and **(J)** *p=0.029.

### 3.7 Rubicon reduces the pathogenicity of *Salmonella typhimurium* infection

Intestinal dysbiosis and increased gut permeabilization are well characterized risk factors for increased pathogenicity of enteric infections. Furthermore, Rubicon and CASM have been previously reported to be required for the effective clearance of *Salmonella* via macrophage engulfment and degradation (74). Since we observe altered intestinal epithelial cell architecture leading to dysbiosis and gut leakage, we were interested in understanding the interplay of Rubicon in dissemination and lethality during *Salmonella* infection. We pretreated mice with streptomycin and after 24 hours, orally infected them with 1x10^9^ colony forming units (CFU)/mL *S.t*. In the WT animals, the mice reached an endpoint by day 4, while the Rubcn^-/-^ mice began reaching endpoint at 3 DPI, and continued reaching endpoint at an accelerated rate compared to WT mice (**Figure 7A**).

**Figure 7:**
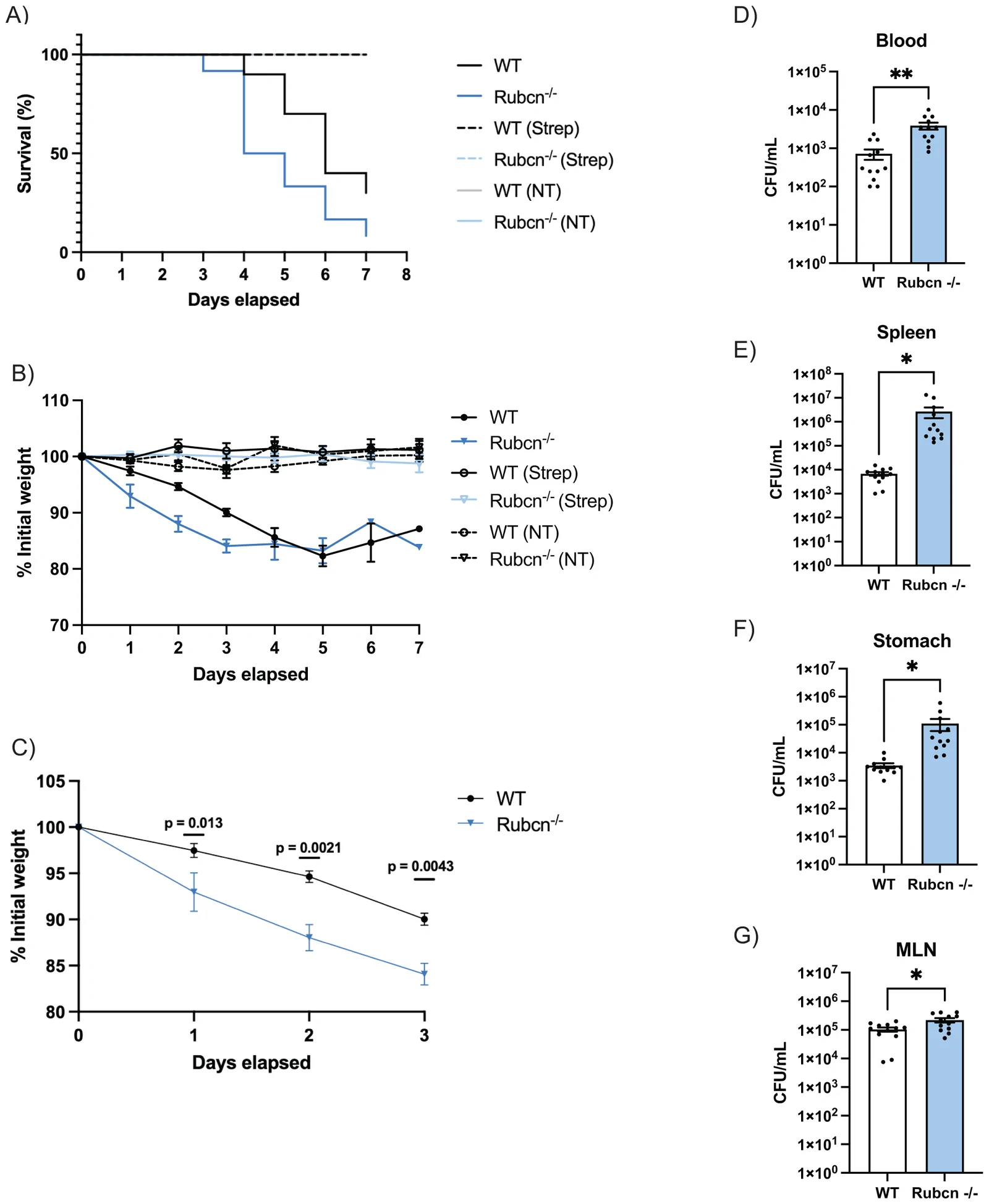
Rubicon limits lethality and systemic dissemination of *Salmonella Typhimurium.* **(A)** Survival curve of 1x10^9^ *S.t* infected 12-week-old WT and Rubcn^-/-^ mice. *n=12* mice for WT infected and Rubcn^-/-^ infected. *n* = 8/group for all other groups. **(B)** Body weight measured as a percentage of initial starting weight of WT and Rubcn^-/-^ mice during infection with *S.t* taken daily. Days 5–7 include mice that had not reached endpoint yet. *n=12* mice for WT infected and Rubcn^-/-^ infected. *n* = 8/group for all other groups. **(C)** Comparison of D1–3 body weights of WT and Rubcn^-/-^ mice during infection with 1x10^9^ *S.t* taken daily. Significance was calculated using a two-tailed unpaired Student’s t-test. (**D–G**) CFU/mL quantification was measured at 3 DPI in a matched infection cohort of 12 animals per genotype. CFU/mL was measured in **(D)** blood, **(E)** spleen, **(F)** stomach, and **(G)** mesenteric lymph nodes (MLN) from mice infected with 10^9^ S.t. Data are represented as mean ± SEM. Significance was calculated using a two-tailed unpaired Student’s t-test. For **(D)** **p=0.0011, **(E)** *p=0.0471, and **(F)** *p=0.0469.

Consistent with Rubcn-deficient infections in zebrafish (71, 75), our results revealed increased and accelerated lethality in the Rubcn^-/-^ mice. Rubcn^-/-^ mice also displayed reductions in body weight that superseded those observed in WT animals (**Figure 7B**), suggesting a more aggressive infection rate. It was our assumption that due to impaired barrier integrity, the Rubcn^-/-^ mice would present with increased bacterial dissemination after 3 days of infection, which contributes to the observed increase in lethality.

To further explore this, we infected streptomycin-pretreated mice with 1x10^9^ CFU/mL *S.t*. We collected blood and organs 3 days post infection (DPI) and assessed the CFU/mL. Again, we could observed significant loss of body weight in the Rubcn^-/-^ mice across the 3 DPI (**Figure 7C**). While we did not detect any significant change in pathogen burden within the majority of peripheral organs of the Rubcn^-/-^ (**Supplementary Figure S6**), we did observe significant increases in bacterial burden within the blood, spleen, and stomach (**Figures 7D–F**) and a trending increase in liver (**Supplementary Figure S6I**), suggesting a more septic phenotype (**Figures 5D, E**). The notion of altered intestinal permeability and entrance of *S.t.* systemically was further supported by increases in bacterial burden observed in the mesenteric lymph nodes (MLN) (**Figure 7G**).

These findings provide novel insight into the role of Rubicon in regulating intestinal barrier integrity as a primary mechanism in preventing bacteremia and lethality during *Salmonella* infection.

## 4 Discussion

### 4.1 Summary of key findings

In this study, we identify Rubicon as an important regulator of intestinal epithelial barrier organization, tissue homeostasis, and protection against enteric infection. Rubcn-/- mice exhibited increased intestinal permeability, elongation of the small and large intestines, altered epithelial architecture, and changes in epithelial proliferation and migration. These phenotypes were accompanied by redistribution of the key adherens-junction protein E-cadherin and the tight-junction protein occludin. At a mechanistic level, Rubcn-/- IECs displayed delayed degradation of internalized cargo and a marked reduction in LC3-positive, occludin-containing vesicles, providing a plausible link between Rubicon-dependent vesicular processing and junctional protein organization. Rubicon-deficiency was also associated with broad changes in the bacterial and viral components of the intestinal microbiome and with increased bacterial burden in the mesenteric lymph nodes (MLNs), blood, spleen, and liver following oral *Salmonella Typhimurium* infection. Together, these findings support a model in which Rubicon-dependent trafficking contributes to epithelial junctional homeostasis and limits the passage and systemic dissemination of enteric pathogens.

### 4.2 Mechanistic links to CASM

Canonical autophagy has established functions in the intestinal epithelium, including maintenance of Paneth cell biology, antimicrobial peptide secretion, epithelial survival, and control of host-microbe interactions (17, 32–34, 36–39, 61, 62). Several of these functions are mediated by proteins, including Rubicon and ATG16L1, that participate in both canonical autophagy and CASM. Our findings suggest that loss of Rubicon produces an intestinal phenotype that is not identical to loss of core autophagy machinery. Rubcn-/- mice did not exhibit a reduction in Paneth cell number or lysozyme abundance, whereas disruption of epithelial ATG16L1 has been associated with Paneth cell abnormalities and impaired antimicrobial function (76, 77). Rubicon-deficiency was also associated with increased intestinal length, rather than the shortening commonly observed in severe experimental intestinal inflammation such as that observed in Crohn’s disease (78, 79). These distinctions argue against interpreting the phenotype as a general consequence of canonical autophagy failure and instead point toward a role for Rubicon-dependent endolysosomal processes in epithelial organization. Nevertheless, because Rubicon has functions beyond CASM and the present study uses a global Rubicon knockout, the observed phenotypes cannot be assigned exclusively to CASM.

The *in vivo* junctional findings provide a potential mechanism for the permeability phenotype. E-cadherin is required for adherens-junction stability, epithelial polarity, and orderly migration along the crypt-villus axis, and intestinal epithelial deletion of E-cadherin disrupts tissue architecture and host defense (80, 81). Occludin is dynamically internalized from the plasma membrane and subsequently recycled or delivered for lysosomal degradation by pathways interacting with or regulated by Beclin 1, making its junctional abundance dependent on appropriate endosomal trafficking (82–84). In Rubcn-/- tissue, total E-cadherin abundance was not overtly reduced, but its spatial distribution was altered, with increased cytosolic E-cadherin and less organized lateral membrane staining. Occludin likewise shifted from a clearly defined peripheral pattern toward a more diffuse intracellular distribution. These results suggest that the barrier defect reflects altered localization and trafficking of junctional proteins rather than simply reduced total protein expression.

The IEC experiments function to further connect this junctional phenotype to Rubicon-dependent vesicular processing. Human IECs have been shown to internalize luminal particles through CASM and to recruit Rubicon and LC3 to cargo-containing compartments before lysosomal processing (65). Consistent with this function, Rubcn-/- IECs exhibited delayed degradation of internalized zymosan. WT IECs also contained abundant LC3-positive vesicles associated with occludin, whereas these structures were strongly reduced in Rubcn-/- IECs. One interpretation is that internalized occludin normally enters a Rubicon-dependent, LC3-positive compartment that contributes to its sorting, recycling, or degradation. Failure to recruit LC3 or properly mature this compartment could lead to intracellular occludin accumulation and disorganized junctional delivery. This model provides a plausible connection among the cargo-processing defect, junctional redistribution, and increased permeability that is observed in the Rubicon-deficient mouse model.

Several features of this proposed mechanism remain to be established. Colocalization of LC3 and occludin does not by itself demonstrate that occludin is enclosed within a single-membrane CASM compartment, nor does it determine whether the relevant trafficking outcome is recycling, degradation, or both. Reduced LC3-occludin overlap in Rubcn-/- cells could reflect defective LC3 recruitment, altered formation or maturation of occludin-containing vesicles, or secondary changes in the abundance and distribution of either protein. Future live-cell pulse-chase studies, surface-labeling assays, and colocalization with early, recycling, late-endosomal, and lysosomal markers will be critical for clarifying the fate of internalized occludin. Ultrastructural analysis or a CASM-selective genetic model, such as disruption of the ATG16L1 WD40-dependent single-membrane lipidation pathway, would more directly establish the identity of these compartments to help refine pathway specificity.

### 4.3 Consequences of the loss of Rubicon-dependent junctional homeostasis

The tissue-level findings are consistent with a chronic defect in epithelial organization. Rubicon-deficient small intestines exhibited shortened villi, lymphoid expansion, epithelial hyperplasia, and disordered stacking of epithelial cells, whereas the colon exhibited elongated crypts and increased inflammatory cell infiltration. BrdU labeling showed increased numbers and migration distances of proliferating cells at 24 hours, with increased migration persisting at 48 hours. These changes may represent a compensatory response to epithelial stress or ineffective cell retention at junctions. However, the cleaved caspase-3 analysis demonstrated only a nonsignificant increasing trend when normalized to total caspase-3. The current data therefore do not establish increased apoptosis as the cause of enhanced proliferation or migration. Altered adhesion, differentiation, extrusion, inflammatory signaling, or mechanical organization could also contribute. Direct quantification of apoptotic cells by TUNEL or annexin V staining, together with lineage tracing or organoid-based migration assays, will be needed to distinguish among these possibilities.

Rubicon-deficiency was also associated with broad restructuring of the intestinal microbiome, including changes in Prevotella, Bacteroides, Acutalibacter, Oscillibacter, Acetatifactor, and several Firmicutes-associated taxa. Changes were additionally detected in viral assignments, including reduced Betaretrovirus and Gammaretrovirus. These trans-kingdom findings are notable because epithelial permeability, antimicrobial signaling, nutrient availability, and cell turnover can all influence the ecological conditions encountered by the intestinal microbiota. Conversely, altered bacterial metabolites and inflammatory signals can further impair junctional integrity. The present data are therefore compatible with a bidirectional relationship in which epithelial dysfunction and microbial imbalance reinforce one another. They do not, however, establish whether microbiome alterations initiate the barrier phenotype or arise as its consequence. The biological significance and source of the altered retroviral assignments also remain uncertain and should not be inferred from taxonomic abundance alone. Longitudinal sampling, littermate and cage-controlled comparisons, co-housing, fecal microbiota transfer, and functional metagenomic or metabolomic analyses will be needed to establish causality and to determine whether the altered microbial community modifies disease severity.

### 4.4 Rubicon is protective against enteric infection and systemic dissemination

Our *S.t.* infection studies demonstrate a physiologically important consequence of the barrier phenotype observed in the Rubicon-deficient mice. Despite the absence of a broad genotype-dependent increase in intestinal bacterial burden, Rubcn-/- mice developed greater weight loss, accelerated mortality, and increased *S.t.* burden in the MLNs, blood, spleen, and liver. The MLNs are a key draining site for organisms that cross the intestinal mucosa and can also function as a barrier that restricts rapid spread to systemic tissues (85). Increased MLN burden therefore provides an important intermediate between the epithelial phenotype and the elevated systemic burden. In combination with the FITC-dextran and junctional data, this pattern is consistent with increased pathogen translocation or reduced early pathogen containment in Rubcn-/- mice. At the same time, Rubicon-dependent CASM contributes to pathogen handling in myeloid cells, and the global knockout may impair bacterial clearance after organisms have crossed the epithelium (86). The systemic infection phenotype is thus likely to reflect contributions from both epithelial barrier dysfunction and altered immune-cell control. Though beyond the scope of the present study, cell-type-specific deletion and reciprocal bone-marrow chimeras or rescue experiments would help separate these mechanisms.

### 4.5 Beyond infection: broader importance of Rubicon-mediated intestinal homeostasis

The relationship of these findings to human intestinal disease should be interpreted broadly. The ATG16L1 T300A risk allele described earlier, provides an important link between autophagy/CASM-related pathways and Crohn’s disease, and this variant can impair both canonical and noncanonical ATG16L1 functions (23–29). However, the present study did not use an induced inflammatory bowel disease model, examine human tissue, or test whether Rubicon expression or CASM activity is altered in individuals with Crohn’s disease. Moreover, increased permeability, villous blunting, epithelial hyperplasia, inflammatory infiltrates, altered epithelial turnover, and dysbiosis are not specific to Crohn’s disease. The elongation of both the small intestine and colon in Rubcn-/- mice also differs from the intestinal shortening frequently observed in severe experimental colitis (78). Accordingly, Rubcn-/- mice should not be described as recapitulating Crohn’s disease.

Instead, the phenotype is relevant to a wider group of barrier-associated enteropathies. Celiac disease, for example, is characterized by increased small-intestinal permeability, villous blunting, crypt hyperplasia, inflammatory-cell accumulation, and altered expression of epithelial junctional proteins (78, 87). Similar combinations of villous remodeling, epithelial inflammation, and barrier dysfunction can occur in infection-associated enteropathies and environmental enteric dysfunction. These shared features provide useful biological context, but the Rubcn-/- model does not reproduce the gluten-specific, HLA-restricted adaptive immune mechanisms that define celiac disease, just as it does not reproduce the complex genetic, microbial, and immune etiology of inflammatory bowel disease. Our most defensible interpretation is therefore that Rubicon regulates a fundamental epithelial homeostatic pathway whose disruption generates features common to several intestinal pathologies.

## 5 Conclusions

In conclusion, our findings identify Rubicon as a novel regulator of intestinal epithelial junctional organization and barrier function. Loss of Rubicon is associated with reduced LC3 localization to occludin-containing vesicles, redistribution of E-cadherin and occludin, altered epithelial architecture and turnover, microbiome restructuring, and increased systemic dissemination of an enteric pathogen. These results expand the intestinal functions attributed to Rubicon-dependent pathways and support a model in which defective vesicular processing compromises epithelial organization and host defense. More broadly, the study provides a mechanistic framework for investigating Rubicon and CASM-associated trafficking across intestinal disorders characterized by epithelial barrier dysfunction, without assigning the phenotype to a single disease entity.

## Data Availability

The data presented in the study are deposited in the Sequence Read Archive (SRA) repository, BioProject ID PRJNA1538719. All other data are available in the manuscript.
