# Supplementary material for: Rubicon is a critical regulator of immune barrier integrity and limits enteric pathogen infection
Source: Front Immunol. 2026 Oct 19;17:1889997. doi: 10.3389/fimmu.2026.1889997 (PMC13724405; doi:10.3389/fimmu.2026.1889997)
Supplement: Supplementary file 1 [file DataSheet1.pdf]

SUPPLEMENTARY DATA

Rubicon is a critical regulator of immune barrier integrity and limits enteric pathogen infection

FIGURE S1

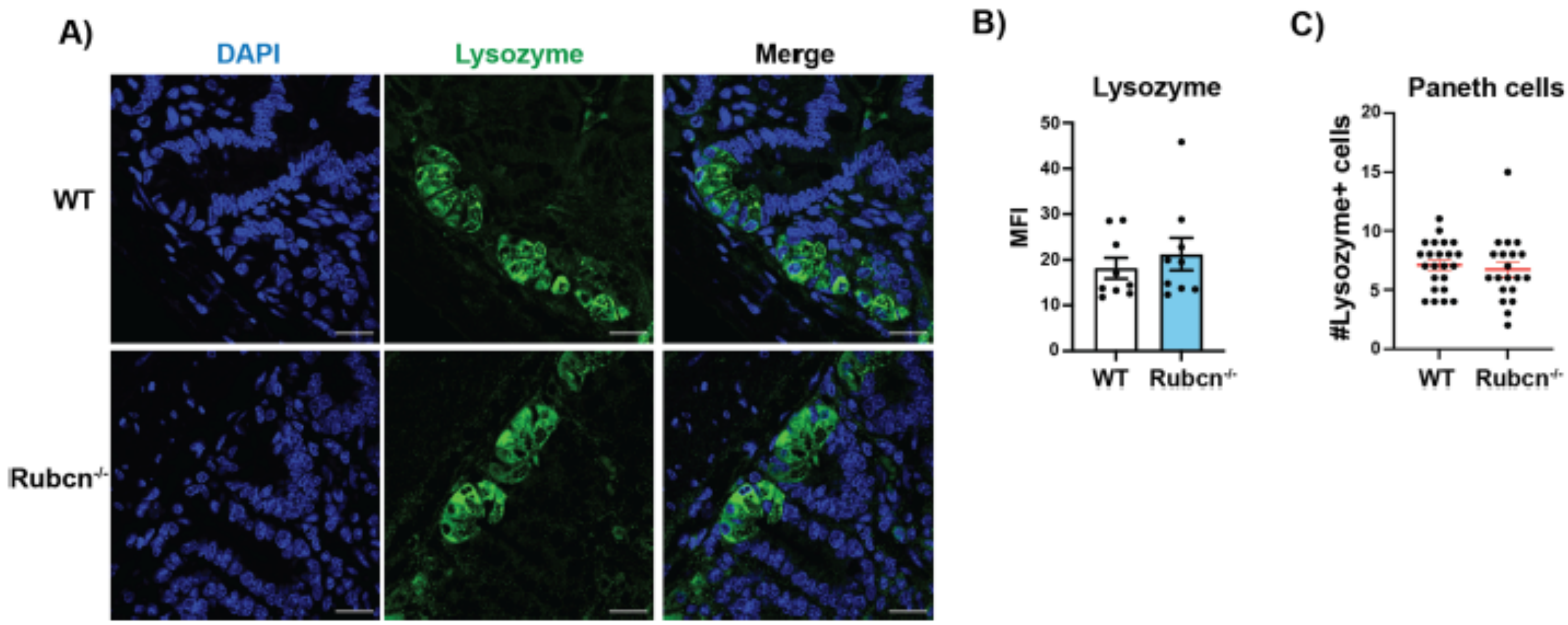

Figure S1: Rubicon-deficiency does not alter Paneth cells.

(**A**) Representative image of lysozyme-positive cells from 10-week-old. Scale bar, 20 μm.

(**B**) Quantification of mean fluorescence intensity (MFI) of lysozyme. Each point represents an image. N = 9 images taken across 3 mice per group. Data are represented as mean ± SEM.

(**C**) Quantification of the number of Paneth cells per crypt. A Paneth cell was defined as lysozyme-positive signal colocalized with DAPI. Each point represents a crypt. Data are represented as mean ± SEM. In reference to Figure 1.

FIGURE S2

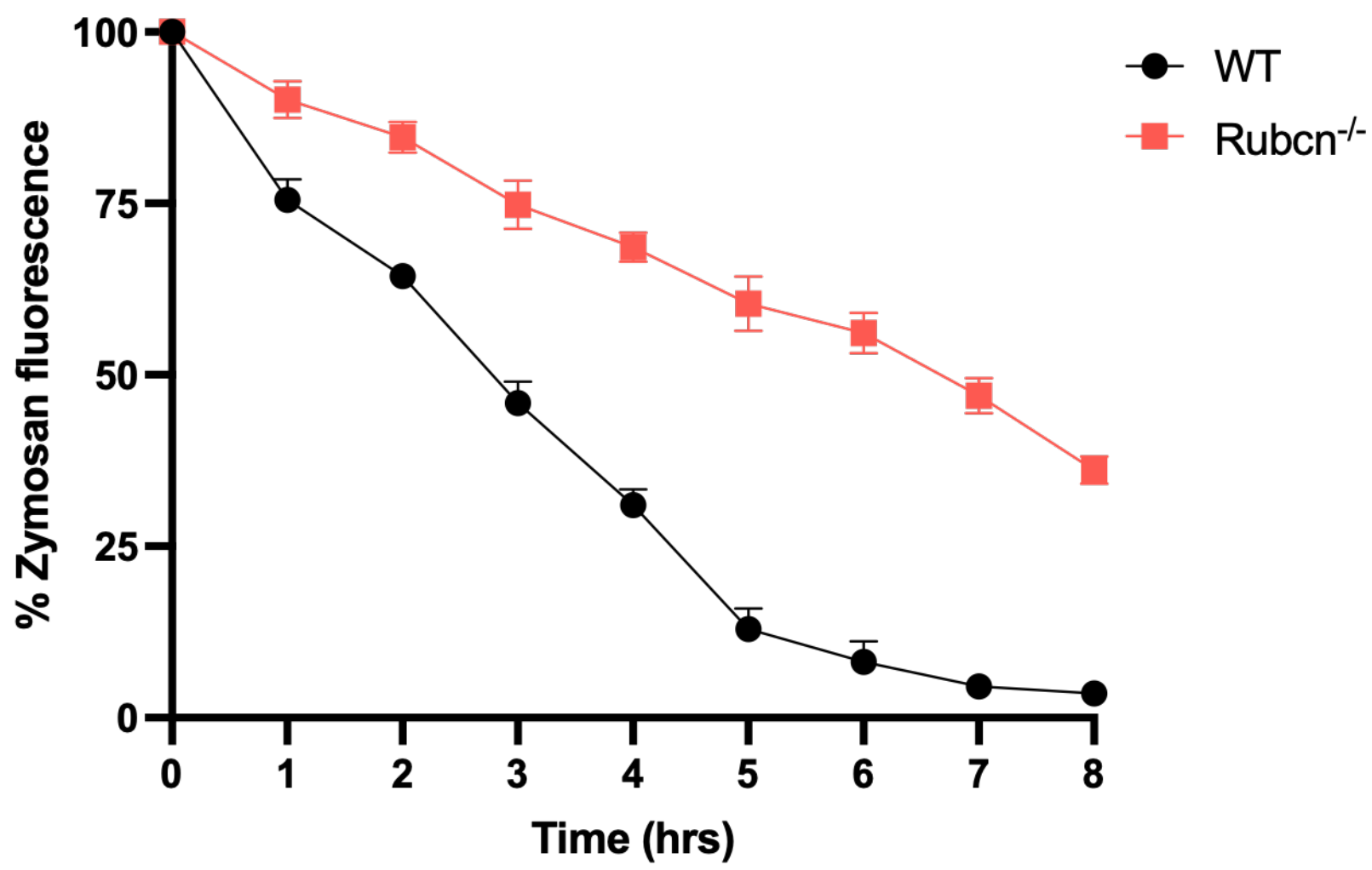

Figure S2: Rubicon-deficiency impairs IEC phagocytosis and vesicle trafficking.

AF red-Labeled zymosan degradation in WT and Rubcn$^{-/-}$ IECs. Data are represented as mean ± SEM, performed in triplicate. In reference to Figure 3.

FIGURE S3

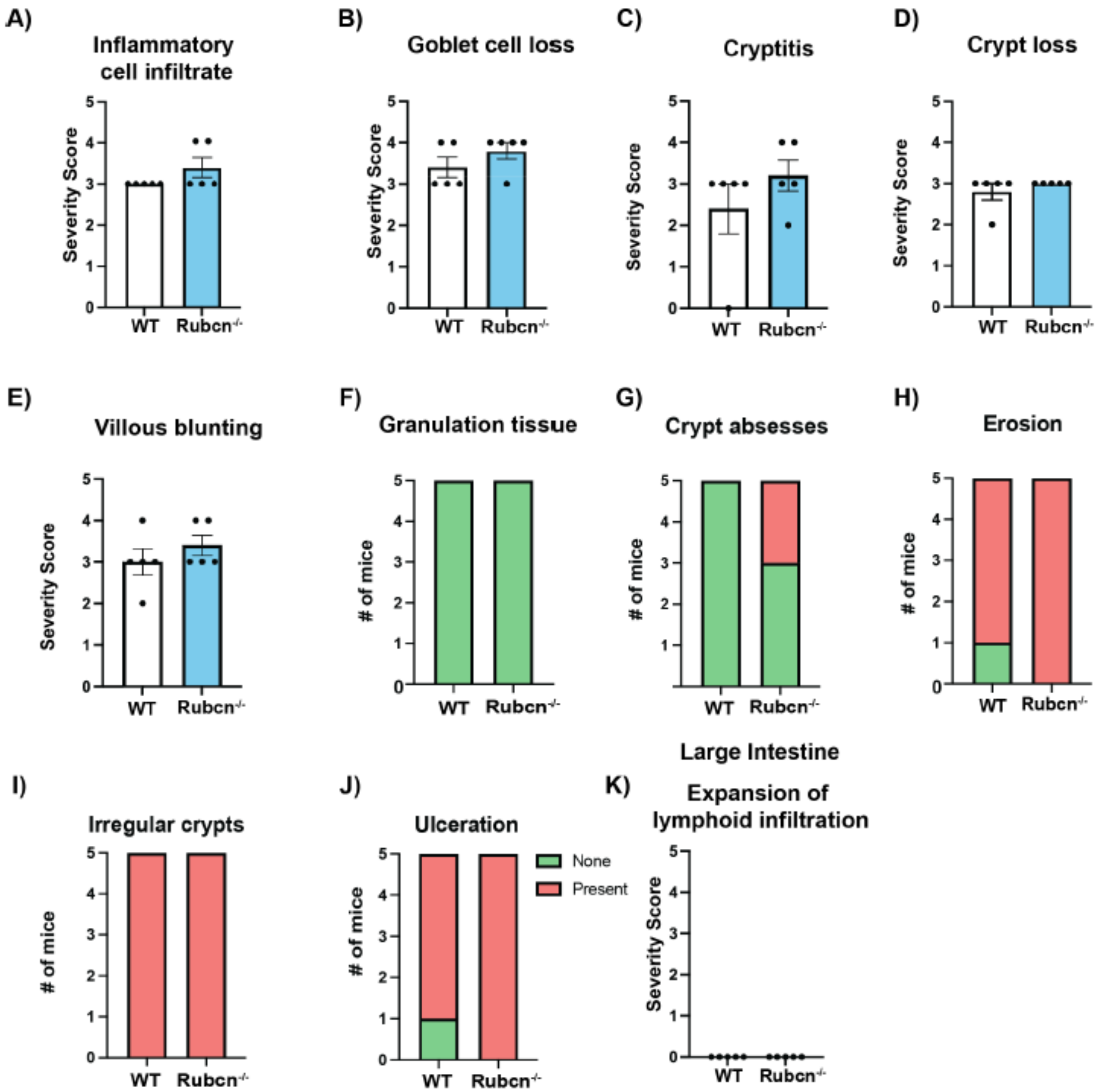

Figure S3: Histological analyses not altered by Rubicon-deficiency

(A-J) Histological quantification of (A) inflammatory cell infiltrate (B) goblet cell loss (C) cryptitis, (D) crypt loss, (E) villous blunting, (F) granulation tissue, (G) crypt abscesses, (H) erosion, (I) irregular crypts, and (J) ulceration within the small intestine of 10-week-old WT and Rubcn-/-. *n* = 5 per group. Each point represents a mouse.
(K) Histological quantification of expansion of lymphoid cells within the large 35 intestine. N = 5 per group. Each point represents a mouse.

Data are represented as mean ± SEM. Severity scores are detailed as follows: 0 = no present, 1 = minimal, 2 = mild, 3 = moderate, 4 = marked. In reference to Figure 4.

FIGURE S4

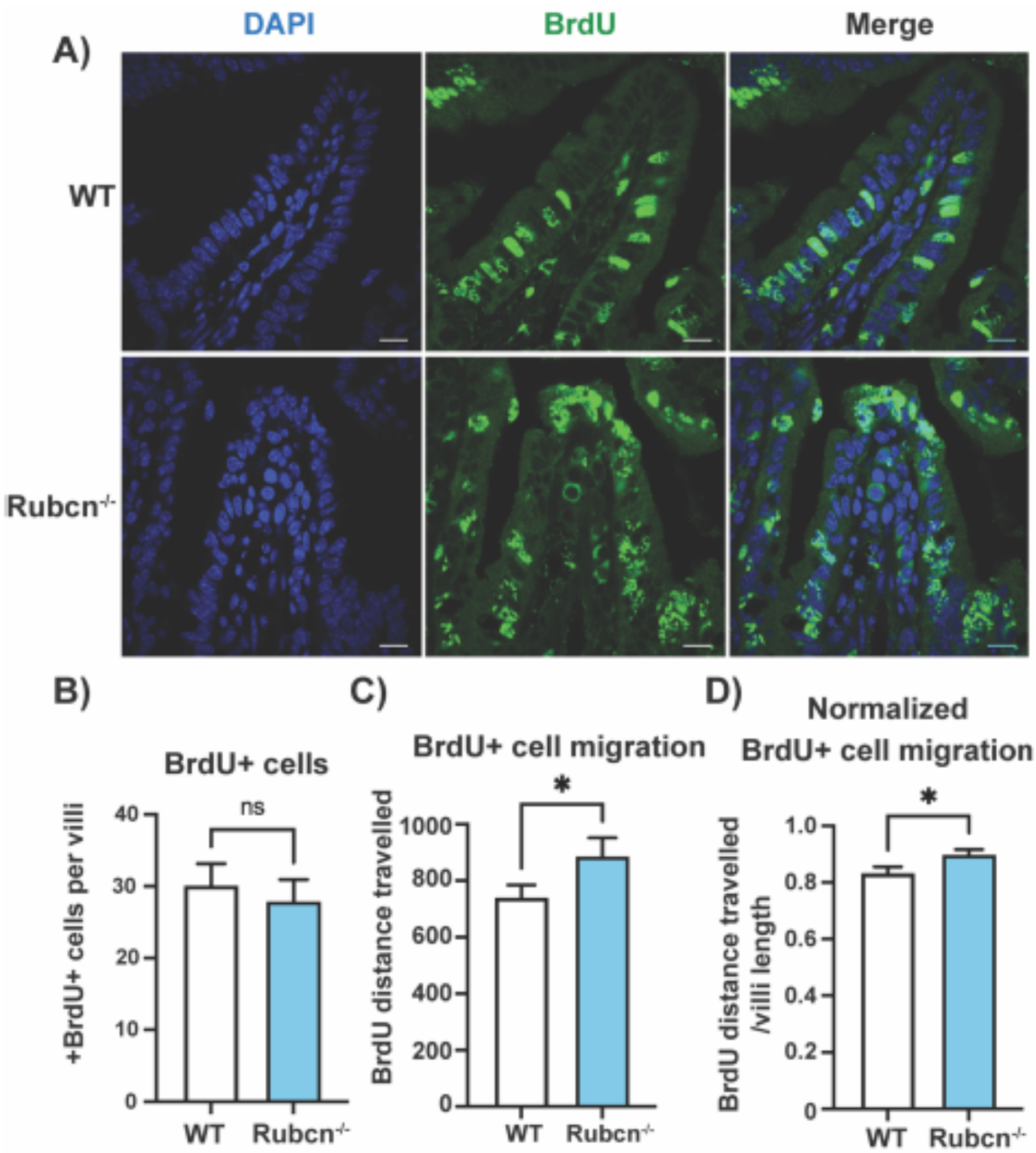

Figure S4: Rubicon-deficiency does not lead to a alter the number of BrdU+ cells

(A) Representative imaging of BrdU-incorporation in the ileum of WT and Rubcn-/- 10-week-old mice. Scale bar, 20 μm.

(B) Quantification of the number of BrdU-postitive cells at 48-hours in the ileum of WT and Rubcn-/- intestines, in reference to Figure 5. Data are represented as mean ± SEM.

(C) Quantification of the number of BrdU-positive cells per villi in each image. *n* = 7 mice per group.

(D) Quantification of the absolute distance of the furthest BrdU-positive cell in each image. *n* = 7 mice per group.

(E) Quantification of the distance of the furthest BrdU-52 positive cell in each image normalized to the length of the villi. *n* = 7 mice per group.

In reference to Figure 5.

FIGURE S5

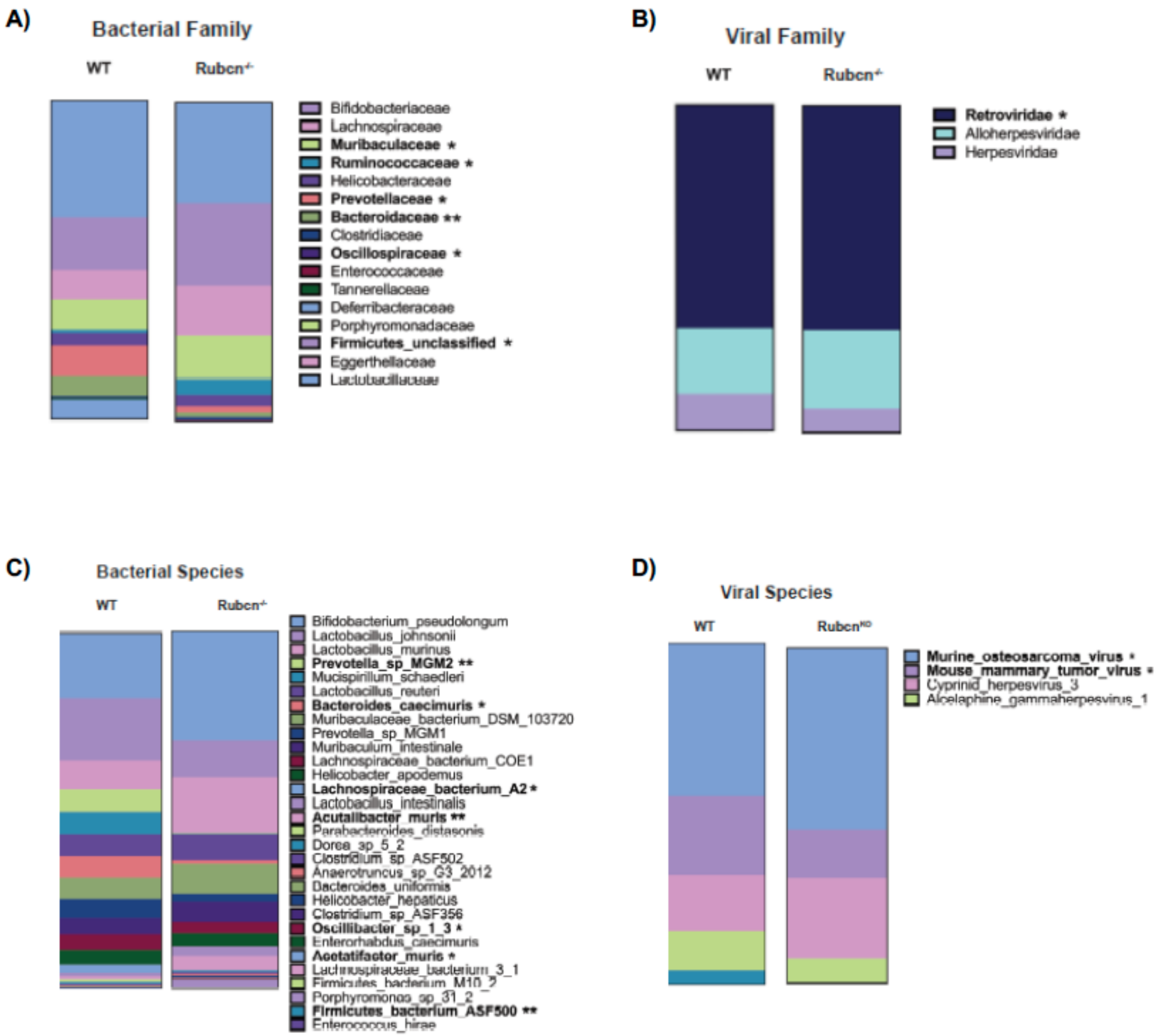

Figure S4: Microbiome analysis for Family and Species Level

(A,B) Family level analysis of bacterial and viral species and (C,D) species level analysis. In reference to Figure 6.

FIGURE S6

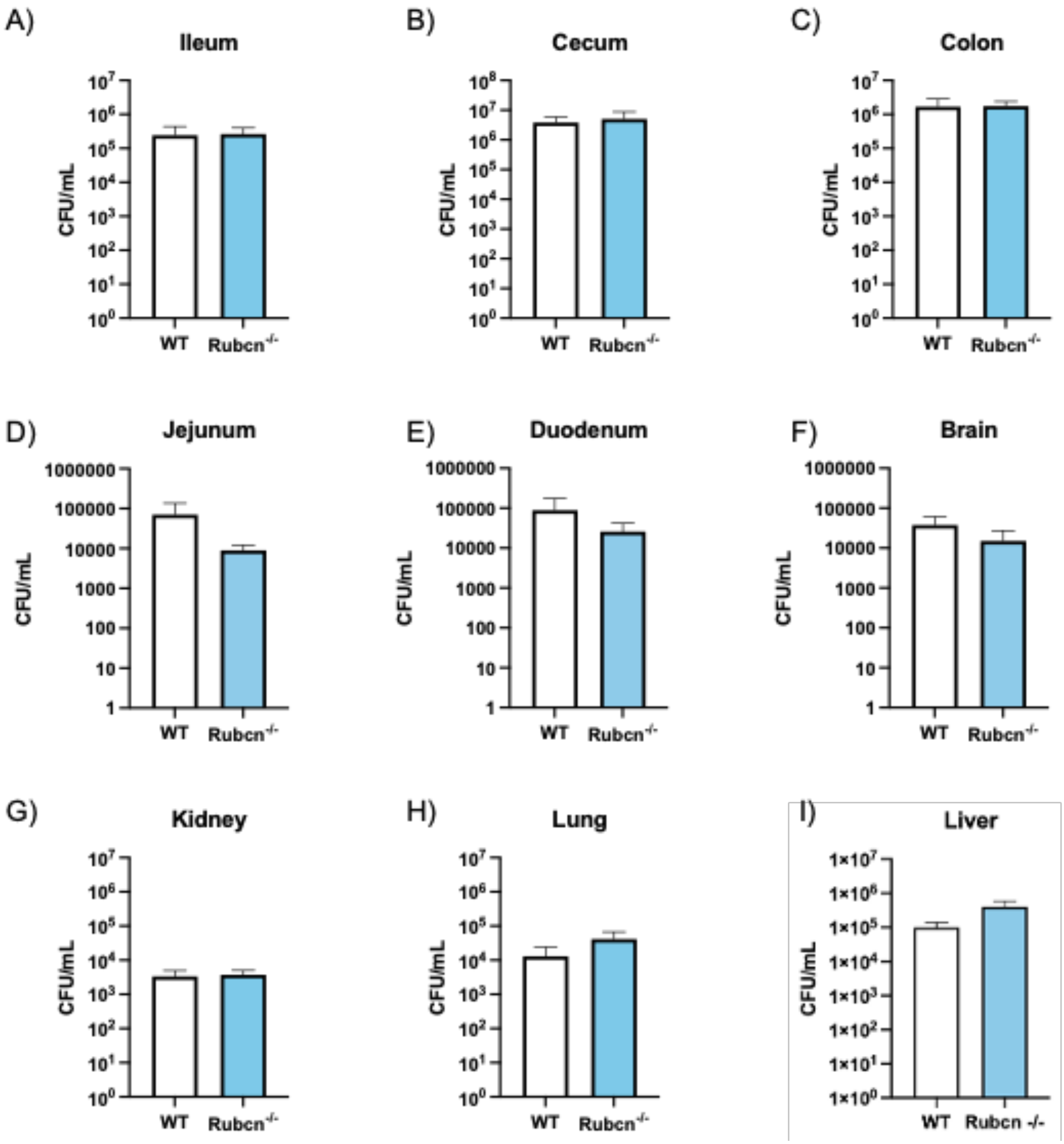

Figure S5: Organs with no significant difference in CFU/mL 3 DPI

(**A-I**) CFU/mL quantification of (A) Ileum, (B) cecum, (C) colon, (D) jejunum, (E) duodenum, (F) brain, (G) kidney, (H) lung, (I) liver in WT and Rubcn-/- 109 S.t infected mice. Organs were collected 3 DPI. In reference to Figure 7.
